# Nailfold capillaroscopy in patients with systemic sclerosis-associated interstitial lung disease: a substudy of the SENSCIS trial

**DOI:** 10.1136/rmdopen-2025-005704

**Published:** 2025-10-17

**Authors:** Vanessa Smith, Christopher P Denton, Ariane L Herrick, Carina Ittrich, Margarida Alves, Maurizio Cutolo

**Affiliations:** 1 Ghent University Hospital, Department of Rheumatology, Ghent University, Department of Internal Medicine and Unit for Molecular Immunology and Inflammation, VIB Inflammation Research Centre (IRC), Ghent, Belgium; 2 Centre for Rheumatology and Connective Tissue Diseases, University College London Division of Medicine, London, UK; 3 Centre for Musculoskeletal Research, The University of Manchester, Northern Care Alliance NHS Foundation Trust, Manchester Academic Health Science Centre, Manchester, UK, and National Institute for Health and Care Research (NIHR) Manchester Biomedical Research Centre (BRC), Manchester, UK; 4 Boehringer Ingelheim Pharma GmbH & Co. KG, Biberach an der Riss, Germany; 5 Boehringer Ingelheim International GmbH, Ingelheim am Rhein, Germany; 6 Laboratory of Experimental Rheumatology, Division of Rheumatology, Department of Internal Medicine, University of Genova, IRCCS San Martino Polyclinic, Genova, Italy

**Keywords:** Scleroderma, Systemic, Treatment, Autoimmune Diseases, Clinical Trial, Lung Diseases, Interstitial

## Abstract

**Objective:**

To assess microvascular changes in nailfold capillaries in patients with systemic sclerosis-associated interstitial lung disease (SSc-ILD) who received nintedanib or placebo in a sub-study of the SENSCIS trial.

**Methods:**

Nailfold capillaroscopy (NC) was performed at baseline and week 52. In the nintedanib and placebo groups, we measured capillary density (number of capillaries/mm), giant capillaries, abnormal shapes and percentage of fingers with microhaemorrhages. In addition, capillary density was evaluated in patients who did/did not have risk factors for rapid forced vital capacity (FVC) decline at baseline and who did/did not have ILD progression (absolute decline in FVC % predicted >5% or death) from baseline to week 52.

**Results:**

Between baseline and week 52, no notable changes were observed in any NC measurement in the overall placebo or nintedanib groups. In patients with risk factors for rapid FVC decline (n=38), there was a numerical reduction in mean capillary density over 52 weeks with placebo, but it remained stable with nintedanib. Among patients who had ILD progression (n=11), there was a numerical increase in mean capillary density over 52 weeks with nintedanib, but it remained stable with placebo. There were no notable changes in capillary density among patients who did not have risk factors for rapid FVC decline at baseline or ILD progression at week 52.

**Conclusion:**

In a substudy of the SENSCIS trial, numerical differences in changes in capillary density assessed by NC over 52 weeks may suggest a potential effect of nintedanib in patients at risk of ILD progression.

**WHAT IS ALREADY KNOWN ON THIS TOPIC**

- Nintedanib is an inhibitor of tyrosine kinases that has shown anti-inflammatory and anti-fibrotic effects and benefits on microvascular alterations in pre-clinical models.

**WHAT THIS STUDY ADDS**

- Nailfold capillaroscopy data from a substudy of the SENSCIS trial in patients with systemic sclerosis-associated interstitial lung disease (SSc-ILD) may suggest a potential effect of nintedanib on microcirculation in patients at risk of ILD progression.

**HOW THIS STUDY MIGHT AFFECT RESEARCH, PRACTICE OR POLICY**

- The utility of nailfold capillaroscopy as a tool for predicting and assessing disease progression in patients with SSc requires further research.

## Introduction

 The pathophysiology of systemic sclerosis (SSc) is a progressive self-amplifying process involving microvascular injury, immune-inflammatory reactions and fibrosis.^1^ Microvascular injury is responsible for manifestations such as Raynaud’s phenomenon and capillary loss and is associated with damage to endothelial cells.^1^ Vascular endothelial growth factor (VEGF) is elevated in the skin and blood of patients with SSc. Recent evidence has suggested that certain VEGF isoforms are anti-angiogenic and may contribute to capillary loss and tissue ischaemia.^2^

Nintedanib is an inhibitor of tyrosine kinases, including the VEGF receptor, which has shown anti-inflammatory and anti-fibrotic effects in preclinical models.^3^ In a mouse model of SSc, nintedanib showed beneficial effects on microvascular alterations, including inhibition of proliferation of pulmonary vascular smooth muscle cells, prevention of thickening of pulmonary vessel walls, inhibition of apoptosis of endothelial cells and a reduction in capillary loss.^4^ Data from the randomised placebo-controlled SENSCIS trial showed that nintedanib slowed the progression of interstitial lung disease associated with SSc (SSc-ILD).^5 6^

Nailfold capillaroscopy (NC) is a non-invasive tool used to evaluate microvascular abnormalities in the nailfolds of the fingers.^7^ A reduction in capillary density assessed by NC has been associated with progression of organ damage in patients with SSc.^8^,^10^ Certain drugs have been hypothesised to influence the progressive obliteration of microvasculature in patients with SSc.^11 12^ In this analysis, we used data from a substudy of the SENSCIS trial to assess microvascular changes in nailfold capillaries as assessed by NC in patients with SSc-ILD who received nintedanib or placebo.

## Methods

### SENSCIS trial and substudy

The design of the SENSCIS trial (NCT02597933) has been published.^5^ Briefly, patients had SSc with first non-Raynaud symptom in the prior ≤7 years, an extent of fibrotic ILD on high-resolution CT≥10%, forced vital capacity (FVC) ≥40% predicted and diffusion capacity of the lung for carbon monoxide 30%–89% predicted. Patients on prednisone ≤10 mg/day or equivalent and/or stable therapy with mycophenolate or methotrexate for ≥6 months were allowed to participate. Patients were randomised to receive nintedanib or placebo, stratified by the presence of anti-topoisomerase I antibody (ATA).

In a sub-study of the SENSCIS trial, NC was performed at baseline and weeks 12, 24, 36 and 52. Images from all eight fingers were assessed by a blinded central reader. Each site used its own nailfold capillaroscope. Patients who opted to participate in the substudy signed a dedicated informed consent form.

### Analyses

Among patients who participated in the substudy, data were summarised by patient for mean capillary density (number of capillaries/mm (linear)), mean number of giant capillaries, mean number of abnormal shapes and percentage of fingers with microhaemorrhages at baseline and week 52. We also evaluated post hoc mean capillary density at baseline and week 52 in (1) patients with and without risk factors for rapid FVC decline (<18 months since first non-Raynaud symptom, elevated inflammatory markers (C-reactive protein≥6 mg/L and/or platelets≥330 x 10^9^ /L) or modified Rodnan skin score (mRSS) >18) at baseline^13^ and (2) patients who did and did not have ILD progression (absolute decline in FVC % predicted >5% or death) from baseline to week 52. Analyses were descriptive. Changes at weeks 12, 24 and 36 are not presented given the changes at week 52 were small.

## Results

Among the patients who consented to participate in the NC substudy (n=120), baseline NC images were not available for 21 patients and were not evaluable for the assessment of capillary density, number of giant capillaries, number of abnormal shapes or fingers with microhaemorrhages for 33–35 patients (online supplemental table 1). At week 52, these numbers were 31 and 24, respectively (online supplemental table 1). Reasons for images being non-evaluable were poor quality images, lack of 1 mm grid or incorrectly placed grid, unclear magnification, and/or wrong device used.

In total, 76 patients provided ≥1 NC measurement at ≥1 visit. These patients came from 21 centres (online supplemental table 2). Among these 76 patients, mean (SD) age was 54.2 (11.9) years, 65.8% were female, 39.5% had diffuse cutaneous SSc, 51.3% were ATA positive, mean mRSS was 10.1 (8.4) and 59.2% were taking mycophenolate. Mean (SD) FVC was 74.2 (17.7) % predicted, and DLco was 50.7 (14.1) % predicted. Baseline characteristics and anti-hypertensive and anti-thrombotic medication use during the trial in the nintedanib and placebo groups are shown in online supplemental tables 3-6.

At baseline, 66, 64, 65 and 65 patients, respectively, had evaluable images to measure capillary density, abnormal shapes, giant capillaries and fingers with microhaemorrhages. At week 52, 65 patients had evaluable images for each of these measurements.

**Figure 1:**
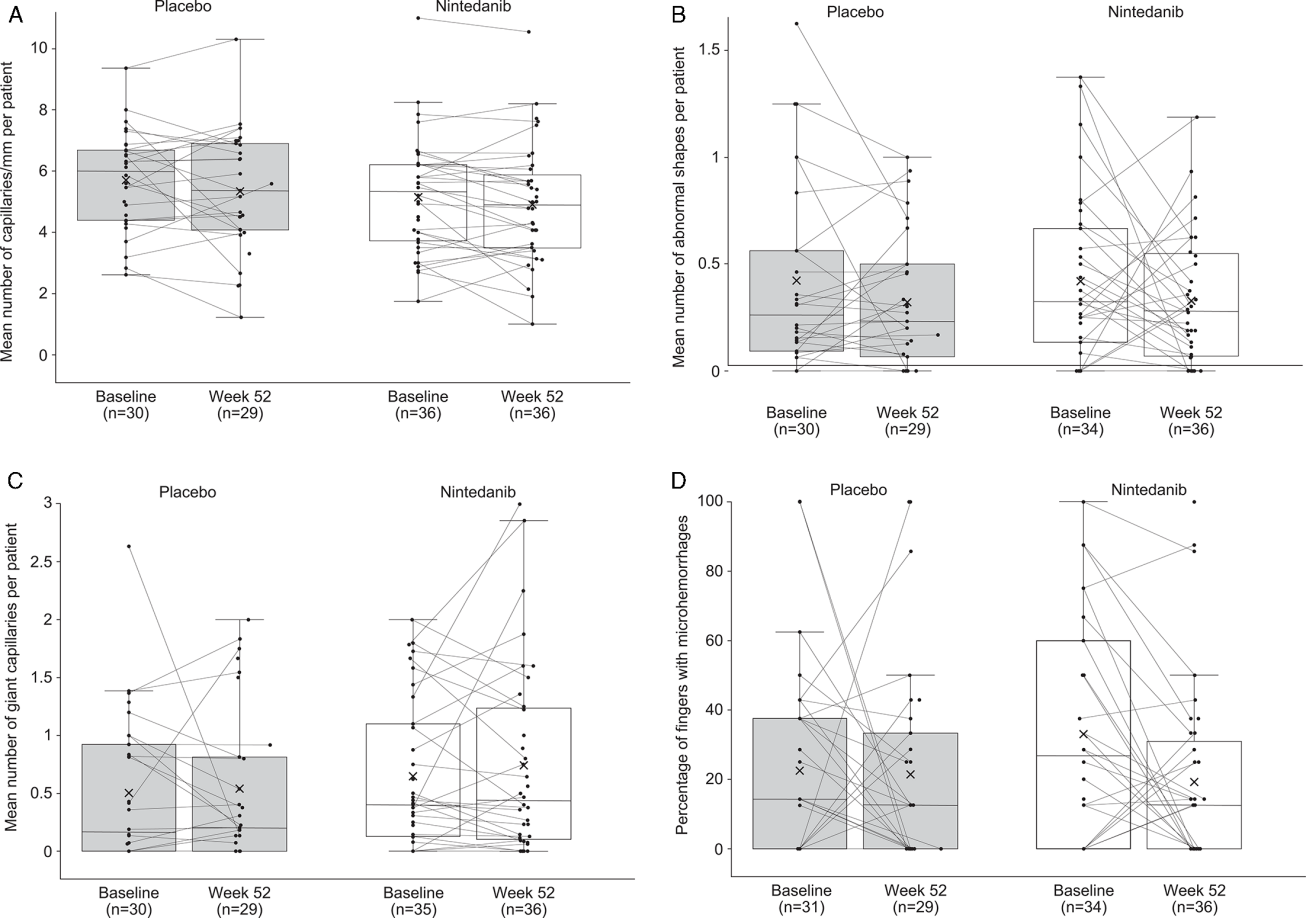
**(A**) Mean capillary density per patient, (**B**) mean number of abnormal shapes per patient, (**C**) mean number of giant capillaries per patient and (D) percentage of fingers with microhaemorrhages at baseline and week 52 in all patients in a substudy of the SENSCIS trial. Markers denote individual measurements, crosses in the boxes mean mid-lines of the boxes medians, boundaries of the boxes 25th and 75th percentiles and whiskers values 1.5× IQR above 75th percentile or below 25th percentile.

At baseline, mean (SD) capillary density per patient was 5.4 (1.8) capillaries/mm, number of abnormal shapes per patient was 0.4 (0.4), number of giant capillaries per patient was 0.6 (0.6) and percentage of fingers with microhaemorrhages was 28.0 (30.4). Between baseline and week 52, no notable changes were observed in any of these measurements in the overall placebo group or the overall nintedanib group (figure 1).

**Figure 2:**
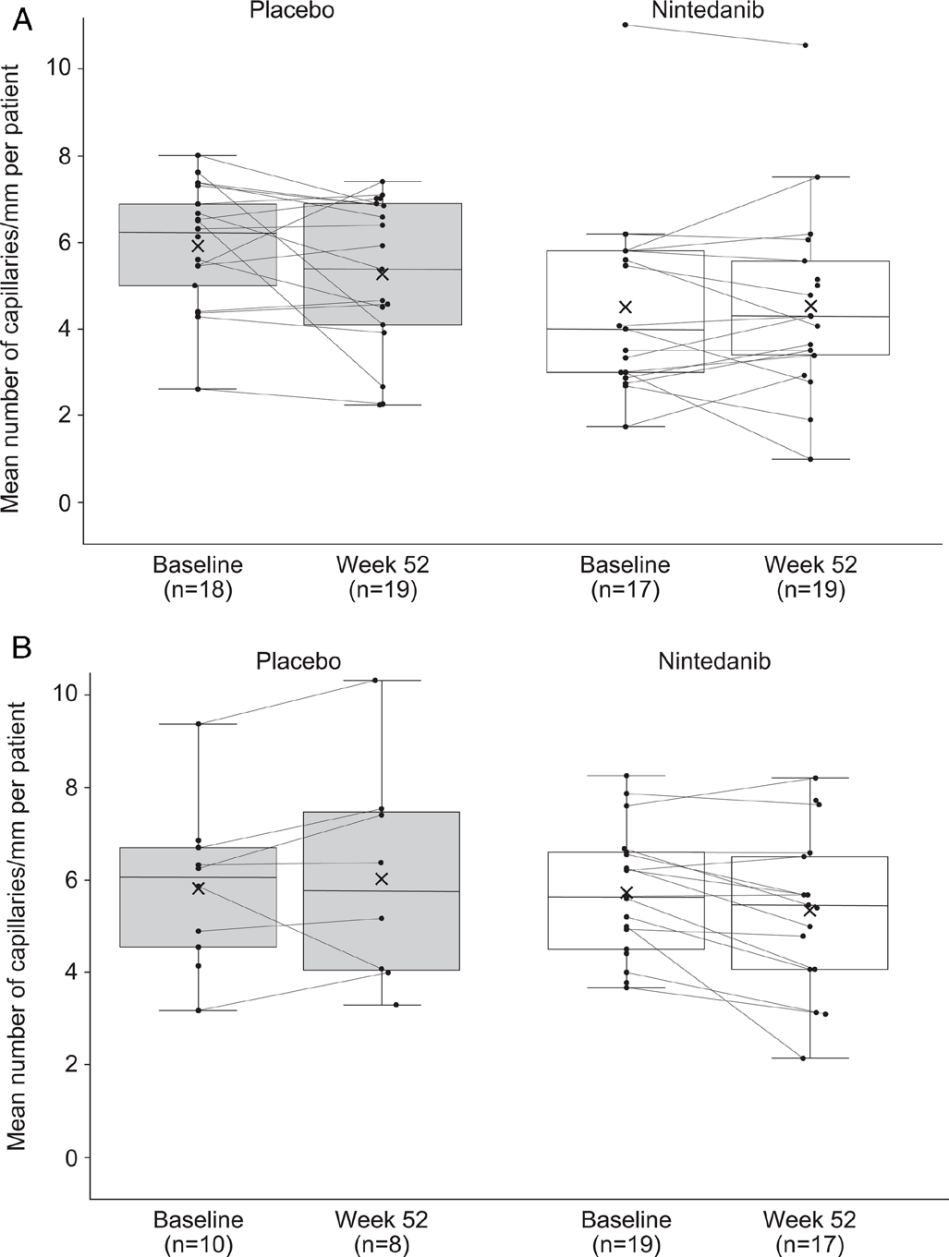
Mean capillary density at baseline and at week 52 in (A) patients with risk factors for rapid FVC decline at baseline and (B) patients without risk factors for rapid FVC decline at baseline in a sub-study of the SENSCIS trial. Markers denote individual measurements, crosses in the boxes mean mid-lines of the boxes medians, boundaries of the boxes 25th and 75th percentiles, and whiskers values 1.5× IQR above 75th percentile or below 25th percentile. FVC, forced vital capacity.

Among patients with and without risk factors for rapid FVC decline at baseline, NC images were evaluable to measure capillary density for 35 and 29 patients at baseline and 38 and 25 patients at week 52, respectively. In patients with risk factors for rapid FVC decline, a numerical reduction in mean capillary density over 52 weeks was observed in the placebo group, but capillary density remained stable in the nintedanib group (figure 2A). There were no notable changes in mean capillary density in either treatment group among patients without risk factors for rapid FVC decline at baseline (figure 2B).

**Figure 3:**
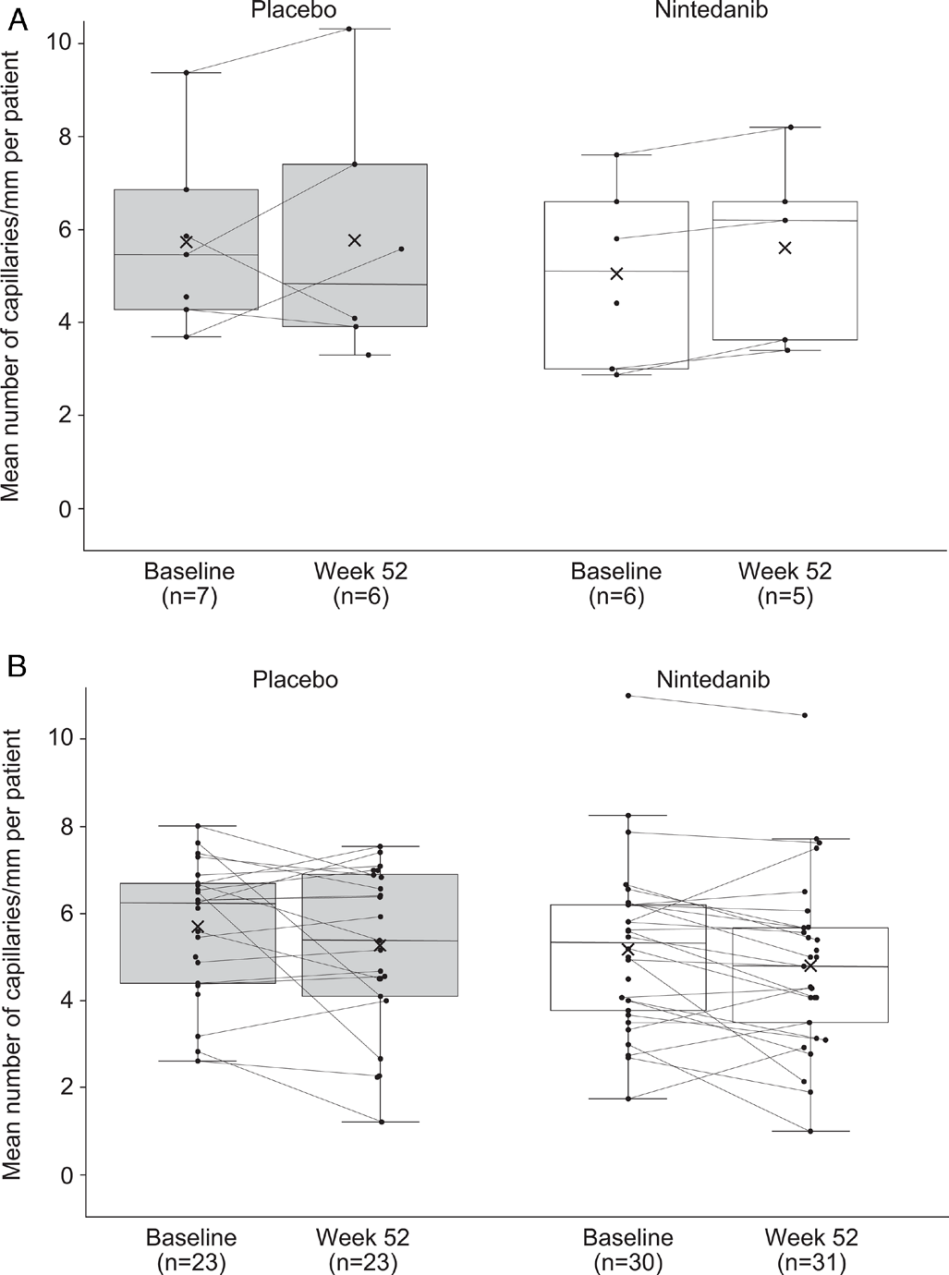
Mean capillary density at baseline and at week 52 in (A) patients who had ILD progression over 52 weeks and (B) patients who did not have ILD progression over 52 weeks in a substudy of the SENSCIS trial. Markers denote individual measurements, crosses in the boxes mean midlines of the boxes medians, boundaries of the boxes 25th and 75th percentiles and whiskers values 1.5× IQR above 75th percentile or below 25th percentile. ILD, interstitial lung disease.

Among patients who had and did not have ILD progression over 52 weeks, NC images were evaluable to measure capillary density for 13 and 53 patients at baseline and 11 and 54 patients at week 52, respectively. Among patients who had ILD progression, a numerical increase in mean capillary density over 52 weeks was observed in the nintedanib group, whereas capillary density remained stable in the placebo group (figure 3A). Among patients who did not have ILD progression, there were no notable changes in capillary density in either treatment group (figure 3B).

## Discussion

In this substudy of the SENSCIS trial in patients with SSc-ILD, no notable changes were observed in microvascular abnormalities based on NC analysis over 52 weeks among patients who received nintedanib or placebo. However, in patients who had risk factors for ILD progression at baseline or who showed ILD progression over 52 weeks, differences in the change in capillary density over 52 weeks may suggest a potential effect of nintedanib. The numerical stabilisation in capillary density in the nintedanib group is interesting given that a serial capillaroscopy study found loss of capillaries to be associated with progression of organ damage, suggesting effects on microcirculation.^8^ However, the effect of nintedanib (and other therapies with presumed microcirculatory effects^11 12^) on microcirculation in patients with SSc needs to be evaluated in dedicated trials. In serial capillaroscopy studies, 49%–76% of patients with SSc did not have significant progression on capillaroscopy.^8 14^ This may in part explain why we only observed a potential effect of nintedanib in patients with risk factors for ILD progression at baseline or ILD progression during the trial.

Several studies have found an association between lower capillary density and lower pulmonary function or the presence of ILD in patients with SSc,^1015^,^17^ but longitudinal data regarding a putative association between loss of capillaries and future progression or development of SSc-ILD are inconclusive.^8 14^ In a study of 140 patients with SSc, loss of capillaries over a 3-year follow-up period was associated with progression of skin fibrosis and peripheral vascular involvement, but not progression of ILD, over the same period,^8^ while in a study in 34 patients with SSc, progressive loss of capillaries (progression to a worse capillaroscopy pattern) over 12 years was associated with a higher prevalence of lung involvement at the end of follow-up.^14^ A study in 334 patients with SSc found that capillaroscopy patterns with lower density at baseline (active and late) were associated with overall disease progression over 2 years, but could not attest an association with new ILD or progression of ILD.^10^

Although further research is required, NC shows promise as a tool in the assessment or prediction of progression of organ manifestations of SSc.^10 18^ NC does not require lengthy training, but the equipment has a relatively high cost and at present its use is mainly restricted to specialist centres.^7^ Various techniques, definitions and scoring systems may be used, but consensus statements from the European Alliance of Associations for Rheumatology and the Scleroderma Clinical Trials Consortium (SCTC) have been developed to standardise and validate procedures and reporting.^7 19^

Our study has limitations. Only a small number of patients provided NC data at baseline and week 52, and NC data were not available from all these patients at both timepoints. As in other multicentre studies, central NC reading was performed by only one reader, a capillaroscopic expert. A 52-week period may have been inadequate to ascertain changes in microvascular abnormalities. In studies that assessed the effects of treatments on capillary number in patients with SSc over ≥3 years, a progressive increase in capillary number (or decrease in capillary loss) was observed each year.^20 21^

In conclusion, in a substudy of the SENSCIS trial in patients with SSc-ILD, numerical differences in changes in capillary density assessed by NC over 52 weeks may suggest a potential effect of nintedanib in patients who had risk factors for ILD progression at baseline or ILD progression during the study. Further research is investigating the utility of NC as a tool for predicting and assessing disease progression in patients with SSc.

## Supplementary material

10.1136/rmdopen-2025-005704 online supplemental file 1

## References

[1] Cutolo M, Soldano S, Smith V (2019). Pathophysiology of systemic sclerosis: current understanding and new insights. Expert Rev Clin Immunol.

[2] Flower VA, Barratt SL, Ward S (2019). The Role of Vascular Endothelial Growth Factor in Systemic Sclerosis. Curr Rheumatol Rev.

[3] Wollin L, Distler JHW, Redente EF (2019). Potential of nintedanib in treatment of progressive fibrosing interstitial lung diseases. Eur Respir J.

[4] Huang J, Maier C, Zhang Y (2017). Nintedanib inhibits macrophage activation and ameliorates vascular and fibrotic manifestations in the Fra2 mouse model of systemic sclerosis. Ann Rheum Dis.

[5] Distler O, Highland KB, Gahlemann M (2019). Nintedanib for Systemic Sclerosis-Associated Interstitial Lung Disease. N Engl J Med.

[6] Assassi S, Distler O, Allanore Y (2022). Effect of Nintedanib on Progression of Systemic Sclerosis-Associated Interstitial Lung Disease Over 100 Weeks: Data From a Randomized Controlled Trial. *ACR Open Rheumatol*.

[7] Smith V, Herrick AL, Ingegnoli F (2020). Standardisation of nailfold capillaroscopy for the assessment of patients with Raynaud’s phenomenon and systemic sclerosis. Autoimmun Rev.

[8] Avouac J, Lepri G, Smith V (2017). Sequential nailfold videocapillaroscopy examinations have responsiveness to detect organ progression in systemic sclerosis. Semin Arthritis Rheum.

[9] Pizzorni C, Sulli A, Paolino S (2017). Progression of Organ Involvement in Systemic Sclerosis Patients with Persistent “Late” Nailfold Capillaroscopic Pattern of Microangiopathy: A Prospective Study. J Rheumatol.

[10] Vanhaecke A, Cutolo M, Distler O (2022). Nailfold capillaroscopy in SSc: innocent bystander or promising biomarker for novel severe organ involvement/progression?. Rheumatology (Oxford).

[11] Smith V, Pizzorni C, Riccieri V (2016). Stabilization of Microcirculation in Patients with Early Systemic Sclerosis with Diffuse Skin Involvement following Rituximab Treatment: An Open-label Study. J Rheumatol.

[12] Wildt M, Andréasson K, Hamberg V (2024). Treatment with mycophenolate mofetil is associated with improved nailfold vasculature in systemic sclerosis. Rheumatology (Oxford).

[13] Khanna D, Maher TM, Volkmann ER (2023). Effect of nintedanib in patients with systemic sclerosis-associated interstitial lung disease and risk factors for rapid progression. RMD Open.

[14] Sulli A, Paolino S, Pizzorni C (2020). Progression of nailfold capillaroscopic patterns and correlation with organ involvement in systemic sclerosis: a 12 year study. Rheumatology (Oxford).

[15] Castellví I, Simeón-Aznar CP, Sarmiento M (2015). Association between nailfold capillaroscopy findings and pulmonary function tests in patients with systemic sclerosis. J Rheumatol.

[16] Vilela VS, Vanhaecke A, da Silva BRA (2022). Is There a Link Between Nailfold Videocapillaroscopy and Pulmonary Function Tests in Systemic Sclerosis Patients?: A 24-Month Follow-up Monocentric Study. J Clin Rheumatol.

[17] De Angelis R, Cipolletta E, Francioso F (2024). Low-Carbon Monoxide Diffusing Capacity, Patient-Reported Measures and Reduced Nailfold Capillary Density Are Associated with Interstitial Lung Disease in Systemic Sclerosis. J Pers Med.

[18] Cutolo M, Herrick AL, Distler O (2016). Nailfold Videocapillaroscopic Features and Other Clinical Risk Factors for Digital Ulcers in Systemic Sclerosis: A Multicenter, Prospective Cohort Study. *Arthritis Rheumatol*.

[19] Smith V, Vanhaecke A, Herrick AL (2019). Fast track algorithm: How to differentiate a “scleroderma pattern” from a “non-scleroderma pattern”. Autoimmun Rev.

[20] Cutolo M, Ruaro B, Pizzorni C (2014). Longterm treatment with endothelin receptor antagonist bosentan and iloprost improves fingertip blood perfusion in systemic sclerosis. J Rheumatol.

[21] Trombetta AC, Pizzorni C, Ruaro B (2016). Effects of Longterm Treatment with Bosentan and Iloprost on Nailfold Absolute Capillary Number, Fingertip Blood Perfusion, and Clinical Status in Systemic Sclerosis. J Rheumatol.

