# Supplementary material for: Nailfold capillaroscopy in patients with systemic sclerosis-associated interstitial lung disease: a substudy of the SENSCIS trial
Source: RMD Open. 2025 Oct 17;11(4):e005704. doi: 10.1136/rmdopen-2025-005704 (PMC12542725; doi:10.1136/rmdopen-2025-005704)
Supplement: online supplemental file 1 [file rmdopen-11-4-s001.docx]

**Nailfold capillaroscopy in patients with systemic sclerosis-associated interstitial lung disease: a sub-study of the SENSCIS trial**

**Online Supplementary Material**

**Supplemental Table 1.** Numbers of patients with evaluable, non-evaluable or missing nailfold capillaroscopy images at baseline and week 52 in a sub-study of the SENSCIS trial (n=120).

|  | **Capillary density** | **Abnormal shapes** | **Number of giant capillaries** | **Percentage of fingers with microhaemorrhages** |
| --- | --- | --- | --- | --- |
| **Baseline** |  |  |  |  |
| Evaluable image | 66 | 64 | 65 | 65 |
| Non-evaluable image* | 33 | 35 | 34 | 34 |
| Missing image | 21 | 21 | 21 | 21 |
| **Week 52** |  |  |  |  |
| Evaluable image | 65 | 65 | 65 | 65 |
| Non-evaluable image* | 24 | 24 | 24 | 24 |
| Missing image | 31 | 31 | 31 | 31 |

*Reasons included poor quality images, lack of 1 mm grid or incorrectly placed grid, unclear magnification and/or wrong device used.

**Supplemental Table 2.** Numbers of centres that provided nailfold capillaroscopy (NC) images of patients in a sub-study of the SENSCIS trial.

| **Number of patients with ≥1 NC measurement* at ≥1 visit** | **Number of centres** |
| --- | --- |
| 1 | 6 |
| 2 | 2 |
| 3 | 4 |
| 4 | 2 |
| 5 | 1 |
| 6 | 3 |
| 7 | 1 |
| 8 | 2 |

*Capillary density, number of giant capillaries, number of abnormal shapes, and/or percentage of fingers with microhaemorrhages.

**Supplemental Table 3.** Baseline of patients with ≥1 nailfold capillaroscopy (NC) measurement (capillary density, number of giant capillaries, number of abnormal shapes, percentage of fingers with microhaemorrhages) at ≥1 visit in the NC sub-study of the SENSCIS trial.

|  | **Nintedanib (n=40)** | **Placebo (n=36)** |
| --- | --- | --- |
| Age (years) | 53.6 (11.6) | 54.8 (12.4) |
| Female | 26 (65.0) | 24 (66.7) |
| Years since first non-Raynaud symptom | 3.5 (1.5) | 3.6 (1.9) |
| Diffuse cutaneous SSc | 15 (37.5) | 15 (41.7) |
| ANA positive | 27 (67.5) | 26 (72.2) |
| ATA positive | 19 (47.5) | 20 (55.6) |
| ARA positive | 3 (7.5) | 2 (5.6) |
| ACA positive | 2 (5.0) | 2 (5.6) |
| Extent of fibrotic ILD on HRCT (%)* | 34.6 (19.8) | 35.0 (21.8) |
| FVC (mL) | 2606 (808) | 2773 (937) |
| FVC % predicted | 72.0 (19.5) | 76.8 (15.4) |
| DLco % predicted^†^ | 50.0 (14.2) | 51.5 (14.1) |
| mRSS | 11.3 (9.4) | 8.8 (7.0) |

Data are n (%) or mean (SD). *****Assessed in whole lung to nearest 5% by central review. Pure (non-fibrotic) ground glass opacity was not included. ^†^Corrected for haemoglobin. ACA, anticentromere antibody; ANA, antinuclear antibody; ARA, anti-RNA polymerase III antibody; ATA, anti-topoisomerase 1 antibody; DLco, diffusing capacity of the lung for carbon monoxide; FVC, forced vital capacity; HRCT, high-resolution computed tomography; ILD, interstitial lung disease; mRSS, modified Rodnan skin score; SSc, systemic sclerosis.

**Supplemental Table 4.** Anti-hypertensive and anti-thrombotic medications used during the study (at baseline and up to week 52, or last trial drug intake if earlier) by patients with ≥1 nailfold capillaroscopy (NC) measurement (capillary density, number of giant capillaries, number of abnormal shapes, percentage of fingers with microhaemorrhages) at ≥1 visit in the NC sub-study of the SENSCIS trial.

|  | **Nintedanib (n=40)** | **Placebo (n=36)** |
| --- | --- | --- |
| Anti-hypertensive drugs* | 37 (92.5) | 28 (77.8) |
| Most frequent anti-hypertensive drugs^†^ |  |  |
| Nifedipine | 11 (27.5) | 13 (36.1) |
| Amlodipine | 9 (22.5) | 4 (11.1) |
| Bosentan | 4 (10.0) | 5 (13.9) |
| Anti-thrombotic drugs* | 17 (42.5) | 16 (44.4) |
| Acetylsalicylic acid | 9 (22.5) | 11 (30.6) |

Data are n (%). Information on dosing and reasons patients were taking these medications was not collected. Patients taking high-dose antiplatelet therapy or full-dose therapeutic anticoagulation were not eligible for the SENSCIS trial. *****Customised drug grouping based on WHODrug Global (formerly WHO Drug Dictionary), version September 2024. ^†^Medications taken by >10% of patients in the nintedanib or placebo group.

**Supplemental Table 5.** Baseline characteristics of patients with and without risk factors for rapid FVC decline at baseline and patients who had and did not have ILD progression over 52 weeks in the nailfold capillaroscopy sub-study of the SENSCIS trial.

|  | **Risk factors for rapid FVC decline at baseline** | | | | **ILD progression over 52 weeks** | | | |
| --- | --- | --- | --- | --- | --- | --- | --- | --- |
|  | **Yes** | | **No** | | **Yes** | | **No** | |
|  | **Nintedanib (n=19)** | **Placebo (n=22)** | **Nintedanib (n=21)** | **Placebo (n=12)** | **Nintedanib (n=6)** | **Placebo (n=8)** | **Nintedanib (n=34)** | **Placebo (n=28)** |
| Age (years) | 50.4 (12.6) | 53.3 (13.2) | 56.5 (10.1) | 58.3 (11.4) | 51.8 (17.1) | 57.8 (11.5) | 53.9 (10.7) | 53.9 (12.7) |
| Female | 15 (78.9) | 17 (77.3) | 11 (52.4) | 6 (50.0) | 4 (66.7) | 6 (75.0) | 22 (64.7) | 18 (64.3) |
| Years since first non-Raynaud symptom | 3.5 (1.7) | 3.5 (2.1) | 3.6 (1.3) | 3.4 (1.1) | 3.1 (1.8) | 4.2 (2.0) | 3.6 (1.4) | 3.4 (1.8) |
| Diffuse cutaneous SSc | 10 (52.6) | 10 (45.5) | 5 (23.8) | 3 (25.0) | 2 (33.3) | 4 (50.0) | 13 (38.2) | 11 (39.3) |
| ANA positive | 16 (84.2) | 17 (77.3) | 11 (52.4) | 8 (66.7) | 4 (66.7) | 4 (50.0) | 23 (67.6) | 22 (78.6) |
| ATA positive | 10 (52.6) | 11 (50.0) | 9 (42.9) | 8 (66.7) | 2 (33.3) | 5 (62.5) | 17 (50.0) | 15 (53.6) |
| ARA positive | 1 (5.3) | 2 (9.1) | 2 (9.5) | 0 | 2 (33.3) | 0 | 1 (2.9) | 2 (7.1) |
| ACA positive | 0 | 2 (9.1) | 2 (9.5) | 0 | 0 | 0 | 2 (5.9) | 2 (7.1) |
| Extent of fibrotic ILD on HRCT (%)* | 38.9 (21.8) | 35.5 (23.0) | 30.7 (17.3) | 33.3 (22.2) | 32.5 (16.4) | 38.8 (21.3) | 35.0 (20.5) | 33.9 (22.3) |
| FVC (mL) | 2228 (622) | 2627 (948) | 2947 (817) | 3001 (958) | 2275 (770) | 3114 (866) | 2664 (812) | 2675 (948) |
| FVC % predicted | 64.2 (17.7) | 76.0 (15.0) | 79.0 (18.8) | 78.0 (14.4) | 64.3 (16.4) | 86.5 (17.6) | 73.3 (19.9) | 74.0 (13.9) |
| DLco % predicted^†^ | 46.7 (12.4) | 48.3 (13.2) | 52.9 (15.4) | 57.4 (15.1) | 38.2 (6.1) | 59.4 (20.0) | 52.0 (14.3) | 49.3 (11.4) |
| mRSS | 15.3 (11.0) | 9.9 (7.4) | 7.6 (5.7) | 6.0 (5.4) | 11.3 (15.2) | 10.7 (6.7) | 11.2 (8.3) | 8.3 (7.1) |

Data are n (%) or mean (SD). *****Assessed in whole lung to nearest 5% by central review. Pure (non-fibrotic) ground glass opacity was not included. ^†^Corrected for haemoglobin.. ACA, anticentromere antibody; ANA, antinuclear antibody; ARA, anti-RNA polymerase III antibody; ATA, anti-topoisomerase 1 antibody; DLco, diffusing capacity of the lung for carbon monoxide; FVC, forced vital capacity; HRCT, high-resolution computed tomography; ILD, interstitial lung disease; mRSS, modified Rodnan skin score; SSc, systemic sclerosis.

**Supplemental Table 6.** Anti-hypertensive and anti-thrombotic medications used during the study (at baseline and up to week 52, or last trial drug intake if earlier) by patients with and without risk factors for rapid FVC decline at baseline and patients who had and did not have ILD progression over 52 weeks in the nailfold capillaroscopy sub-study of the SENSCIS trial.

|  | **Risk factors for rapid FVC decline at baseline** | | | | **ILD progression over 52 weeks** | | | |
| --- | --- | --- | --- | --- | --- | --- | --- | --- |
|  | **Yes** | | **No** | | **Yes** | | **No** | |
|  | **Nintedanib (n=19)** | **Placebo (n=22)** | **Nintedanib (n=21)** | **Placebo (n=12)** | **Nintedanib (n=6)** | **Placebo (n=8)** | **Nintedanib (n=34)** | **Placebo (n=28)** |
| Anti-hypertensive drugs* | 17 (89.5) | 17 (77.3) | 20 (95.2) | 10 (83.3) | 6 (100.0) | 6 (75.0) | 31 (91.2) | 22 (78.6) |
| Most frequent anti-hypertensive drugs^†^ |  |  |  |  |  |  |  |  |
| Nifedipine | 6 (31.6) | 9 (40.9) | 5 (23.8) | 4 (33.3) | 2 (33.3) | 1 (12.5) | 9 (26.5) | 12 (42.9) |
| Bosentan | 3 (15.8) | 3 (13.6) | n/a | n/a | 2 (33.3) | 1 (12.5) | 2 (5.9) | 4 (14.3) |
| Amlodipine | 2 (10.5) | 3 (13.6) | 7 (33.3) | 1 (8.3) | 1 (16.7) | 1 (12.5) | 8 (23.5) | 3 (10.7) |
| Anti-thrombotic drugs* | 7 (36.8) | 11 (50.0) | 10 (47.6) | 5 (41.7) | 3 (50.0) | 2 (25.0) | 14 (41.2) | 14 (50.0) |
| Acetylsalicylic acid | 5 (26.3) | 7 (31.8) | 4 (19.0) | 4 (33.3) | 2 (33.3) | 1 (12.5) | 7 (20.6) | 10 (35.7) |

Data are n (%). Information on dosing and reasons patients were taking these medications was not collected. Patients taking high-dose antiplatelet therapy or full-dose therapeutic anticoagulation were not eligible for inclusion in the SENSCIS trial. *****Customised drug grouping based on WHODrug Global (formerly WHO Drug Dictionary), version September 2024. ^†^Medications taken by >10% of patients in the nintedanib or placebo group among those with patients with ≥1 nailfold capillaroscopy measurement at ≥1 visit. n/a: not available (but taken by ≤10% of patients in both arms of this subgroup).
